# Supplementary material for: Analysis of the Changes in Diversity of Culturable Bacteria in Different Niches of Mulberry Fields and Assessment of Their Plant Growth-Promoting Potential
Source: Microorganisms. 2025 Apr 28;13(5):1012. doi: 10.3390/microorganisms13051012 (PMC12114483; doi:10.3390/microorganisms13051012)
Supplement: Supplementary file 1 [file microorganisms-13-01012-s001.zip › microorganisms-3543963-supplementary.pdf]

# Supplementary Materials

Supplementary materials for “Analysis of the Changes in Diversity of Culturable Bacteria in Different Niches of Mulberry Fields and Assessment of Their Plant Growth-Promoting Potential”

**Supplementary Table S1:**

Information on the collection of mulberry rhizosphere soil and root, stem, leaf samples.

| No. | Collection site | Longitude and latitude |
|---|---|---|
| 1 | Jiulong Town, Yingde City, China | E112.92°，N24.11° |
| 2 | Hanguang Town, Yingde City, China | E113.11°，N24.23° |
| 3 | Qigong Town, Yangshan County, China | E112.56°，N24.31° |
| 4 | Zengcheng District, Guangzhou, China | E113.64°，N23.24° |
| 5 | South China Agricultural University, China | E113.37°，N23.16° |
| 6 | Regional Sericulture Training Center for Asia-Pacific | E113.35°，N23.16° |

**Supplementary Table S2:**

Primer sequence information of key genes of antimicrobial synthesis.

| Antibiotic | Genes | Primers | Sequences (5' to 3') | Fragment length/bp |
|---|---|---|---|---|
| Surfactin | *Sfp* | SfpF | ATGAAGATTTACGGAATTTA | 675 |
| | | SfpR | TTATAAAAGCTCTTCGTACG | |
| | *SfrC* | Sur3F | ACAGTATGGAGGCATGGTC | 441 |
| | | Sur3R | TTCCGCCACTTTTTCAGTTT | |
| | *SrfAA* | Srfkn-1 | AGCCGTCCTGTCTGACGACG | 1500 |
| | | Srfkn-2 | TCTGCTGCCATACCGCATCGTC | |
| Iturin | *ItuA* | ituAF | ATGTATACCAGTCAATTCC | 1150 |
| | | ituAR | GATCCGAAGCTGACAATAG | |
| | *ItuC* | ITUC-F | CCCCCTCGGTCAAGTGAATA | 594 |
| | | ITUC-R | TTGGTTAAGCCCTGATGCTC | |
| | *ItuD* | ItuD1F | GATGCGATCTCCTTGGATGT | 647 |
| | | ItuD1R | ATCGTCATGTGCTGCTTGAG | |
| Fengycin | *FenA* | FenAa | AAGAGATTCAGTAAGTGGCCCATCCAG | 1500 |
| | | FenAb | CGCCCTTTGGGAAGAGGTGC | |
| | *FenB* | fenBF | CTATAGTTTGTTGACGGCTC | 1400 |
| | | fenBR | CAGCACTGGTTCTTGTCGCA | |
| | *FenD* | FenD1f | TTTGGCAGCAGGAGAAGTTT | 964 |
| | | FenD1r | GCTGTCCGTTCTGCTTTTTC | |
| Bacillomycin | *bamC* | BAMC-F | AGTAAATGAACGCGCCAATC | 957 |
| | | BAMC-R | CCCTCTCCTGCCACATAGAG | |
| Polyketide | *PKSI* | KSF | GCGATGGATCCNCAGCAGCG | 700 |
| | | KSR | GTGCCGGTNCCGTGNGYYTC | |
| Nonribosomal peptide | *NRPS* | NRPSF | GCNGGYGGYGCNTAYGTNCC | 1000 |
| | | NRPSR | CCNCGDATYTTNACYTG | |

**Supplementary Table S3:**

Distribution and isolation frequency of bacteria in different niches of mulberry fields.

| | Taxa | Isolation frequency (%) | | | |
|---|---|---|---|---|---|
| | | MRSB | MEB | SIB | Total |
| 1 | *Bacillus* spp. | 17.92% | 6.36% | 1.45% | 25.72% |
| 2 | *Pseudomonas* spp. | 6.94% | 11.85% | 1.73% | 20.52% |
| 3 | *Enterobacter* spp. | 2.60% | 7.23% | 10.69% | 20.52% |
| 4 | *Arthrobacter* spp. | 2.02% | 0.29% | 0.00% | 2.31% |
| 5 | *Pantoea* spp. | 1.45% | 2.31% | 0.00% | 3.76% |
| 6 | *Acinetobacter* spp. | 0.87% | 2.89% | 0.00% | 3.76% |
| 7 | *Serratia* spp. | 0.58% | 0.00% | 0.58% | 1.16% |
| 8 | *Brevibacterium* spp. | 0.58% | 0.00% | 0.29% | 0.87% |
| 9 | *Agrobacterium* spp. | 0.58% | 0.00% | 0.00% | 0.58% |
| 10 | *Streptomyces* sp. | 0.29% | 0.00% | 0.00% | 0.29% |
| 11 | *Staphylococcus* spp. | 0.29% | 0.00% | 2.31% | 2.60% |
| 12 | *Lysinibacillus* sp. | 0.29% | 0.00% | 0.00% | 0.29% |
| 13 | *Bordetella* sp. | 0.29% | 0.00% | 0.00% | 0.29% |
| 14 | *Pseudochrobactrum* sp. | 0.29% | 0.00% | 0.00% | 0.29% |
| 15 | *Luteimonas* sp. | 0.29% | 0.00% | 0.00% | 0.29% |
| 16 | *Stenotrophomonas* spp. | 0.29% | 1.45% | 0.29% | 2.02% |
| 17 | *Alcaligenes* sp. | 0.29% | 0.00% | 0.00% | 0.29% |
| 18 | *Cellulosimicrobium* spp. | 0.29% | 0.00% | 0.29% | 0.58% |
| 19 | *Microbacterium* spp. | 0.29% | 0.00% | 0.58% | 0.87% |
| 20 | *Klebsiella* spp. | 0.00% | 0.29% | 0.29% | 0.58% |
| 21 | *Curtobacterium* sp. | 0.00% | 0.29% | 0.00% | 0.29% |
| 22 | *Erwinia* sp. | 0.00% | 0.29% | 0.00% | 0.29% |
| 23 | *Delftia* sp. | 0.00% | 0.29% | 0.00% | 0.29% |
| 24 | *Xanthomonas* sp. | 0.00% | 0.29% | 0.00% | 0.29% |
| 25 | *Leucobacter* sp. | 0.00% | 0.29% | 0.00% | 0.29% |
| 26 | *Enterococcus* spp. | 0.00% | 0.00% | 9.54% | 9.54% |
| 27 | *Micrococcus* spp. | 0.00% | 0.00% | 0.58% | 0.58% |
| 28 | *Aeromonas* sp. | 0.00% | 0.00% | 0.29% | 0.29% |
| 29 | *Brevundimonas* sp. | 0.00% | 0.00% | 0.29% | 0.29% |
| 30 | *Brachybacterium* sp. | 0.00% | 0.00% | 0.29% | 0.29% |
| | total | 36.42% | 34.10% | 29.48% | 100.00% |

MRSB, MEB, and SIB represent the communities isolated from the mulberry rhizosphere soil bacteria, mulberry endophytic bacteria and silkworm intestinal bacteria, respectively.

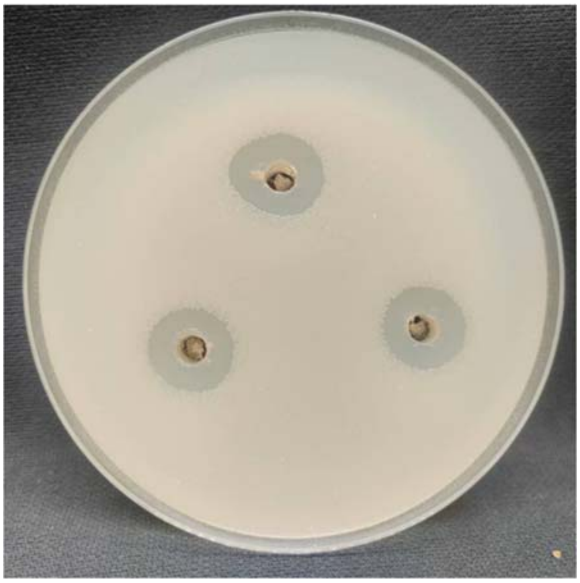

**Supplementary Figure S1:** Detection of the antibacterial activity of the isolated bacterial strains.

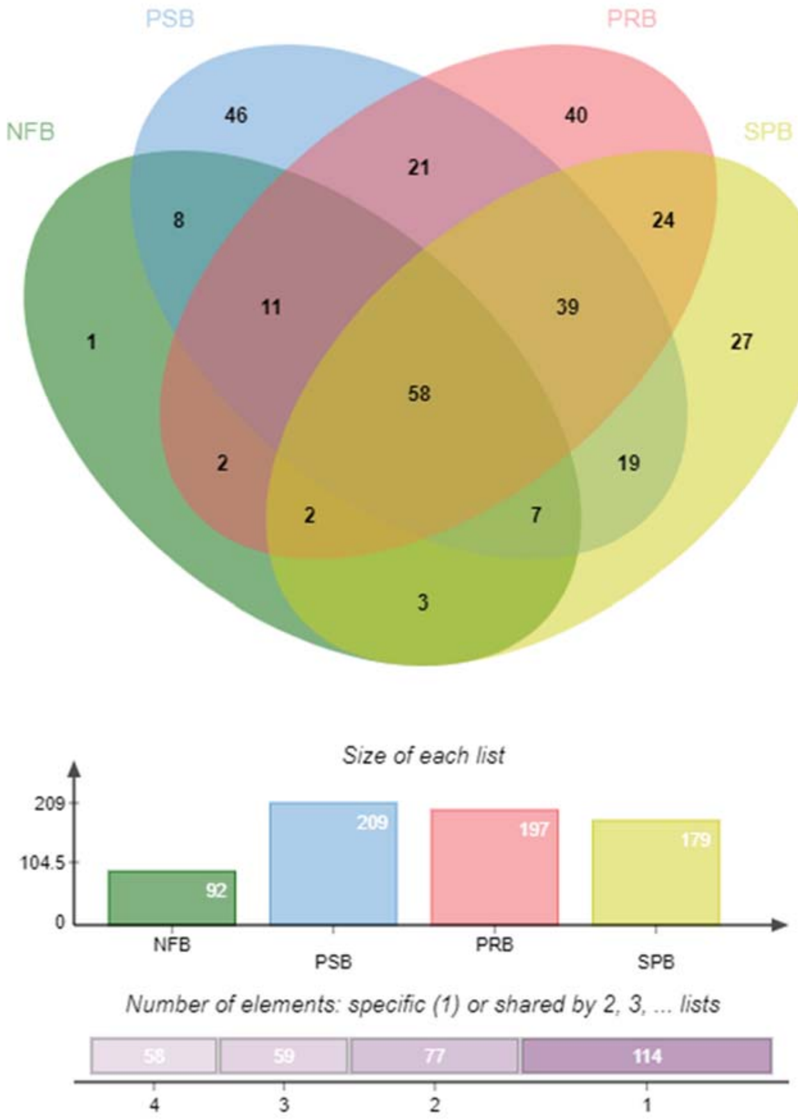


**Supplementary Figure S2:** Venn diagram of nitrogen fixation, phosphate solubilization, potassium release and siderophore production by bacteria. NFB, PSB, PRB, and SPB represent nitrogen - fixing bacteria, phosphate - solubilizing bacteria, potassium - releasing bacteria, and siderophore - producing bacteria, respectively.
